# Do Clinicians Need to Rethink Antipsychotic Maintenance Treatment? Pro

**DOI:** 10.1192/j.eurpsy.2022.43

**Published:** 2022-09-01

**Authors:** J. Kane

**Affiliations:** Northwell Health, Department Of Psychiatry, Zucker Hillside Hospital, Glen Oaks, United States of America

## Abstract

Schizophrenia remains a challenging illness to treat despite considerable progress. The heterogeneity of symptoms and variability in course, outcome, and treatment response lead to considerable uncertainties in treatment guidelines. One of the most challenging issues is how to determine the need for and duration of the maintenance treatment that is intended to reduce the risk of exacerbation, relapse, and hospitalization. It is clear that in large populations, antipsychotic medication is very effective in reducing those risks, however, there is likely a subpopulation of patients with diminished risk for such outcomes even when medication is discontinued. Although attempts have been made to utilize “intermittent” or “targeted” treatment, those randomized controlled trials that have been conducted show a statistically significant advantage for continuous treatment. At the same time, many patients continue to receive higher than necessary doses of antipsychotic medication and practice needs to be reevaluated in terms of dosage requirements. The challenge remains that there are no good biomarkers or predictors of who is at risk and, therefore, dosage reduction is only titrated against exacerbation or relapse. We need to proactively reconsider who requires maintenance treatment by establishing predictive biomarkers (clinical or biological) that can help us to have a more personalized approach.

**Disclosure:**

Consultant to or receives honoraria: Alkermes, Allergan, Dainippon Sumitomo, H. Lundbeck, Indivior, Intracellular Therapies, Janssen Pharmaceutical, Johnson & Johnson, LB Pharmaceuticals, Merck, Minerva, Neurocrine, Novartis Pharmaceuticals,

